# Seasonal and year-round use of the Kushiro Wetland, Hokkaido, Japan by sika deer (*Cervus nippon yesoensis*)

**DOI:** 10.7717/peerj.3869

**Published:** 2017-10-12

**Authors:** Hino Takafumi, Tatsuya Kamii, Takunari Murai, Ryoto Yoshida, Atsuki Sato, Yasuyuki Tachiki, Rika Akamatsu, Tsuyoshi Yoshida

**Affiliations:** 1 Department of Environmental and Symbiotic Sciences, Rakuno Gakuen University, Ebestu, Hokkaido, Japan; 2 EnVision Conservation Office, Sapporo, Hokkaido, Japan; 3 Institute for Tropical Biology and Conservation, University of Malaysia Sabah, Kota Kinabalu, Sabah, Malaysia

**Keywords:** Seasonal migration, Cross-ecosystem movement, Ecosystem maintenance and recovery project, GPS collar, Net squared displacement, Seasonal home range overlap

## Abstract

The sika deer (*Cervus nippon yesoensis*) population in the Ramsar-listed Kushiro Wetland has increased in recent years, and the Ministry of the Environment of Japan has decided to take measures to reduce the impact of deer on the ecosystem. However, seasonal movement patterns of the deer (i.e., when and where the deer inhabit the wetland) remain unclear. We examined the seasonal movement patterns of sika deer in the Kushiro Wetland from 2013 to 2015 by analyzing GPS location data for 28 hinds captured at three sites in the wetland. Seasonal movement patterns were quantitatively classified as seasonal migration, mixed, dispersal, nomadic, resident, or atypical, and the degree of wetland utilization for each individual was estimated. The area of overlap for each individual among intra-capture sites and inter-capture sites was calculated for the entire year and for each season. Our results showed that the movement patterns of these deer were classified not only as resident but also as seasonal migration, dispersal, and atypical. Approximately one-third of the individuals moved into and out of the wetland during the year as either seasonal migrants or individuals with atypical movement. Some of the individuals migrated to farmland areas outside the wetland (the farthest being 69.9 km away). Half of the individuals inhabited the wetland all or most of the year, i.e., 81–100% of their annual home range was within the wetland area. Even among individuals captured at the same site, different seasonal movement patterns were identified. The overlap areas of the home ranges of individuals from the same capture sites were larger than those for individuals from different capture sites (e.g., mean of annual home range overlap with intra-capture sites: 47.7% vs. inter-sites: 1.3%). To achieve more effective ecosystem management including deer management in the wetland, management plans should cover inside and outside of the wetland and separate the population into multiple management units to address the different movement patterns and wetland utilization of the population.

## Introduction

In recent decades, numerous regions in the Northern Hemisphere have experienced increasing cervid populations and expansion of their distributions resulting in significant impacts to natural ecosystems (Côte et al., 2004). Cervids can cause substantial impacts to ecosystem processes and functions through the alteration of plant biomass and community composition (Rooney & Waller, 2003; Côte et al., 2004). These impacts are observed not only in forest ecosystems but also wetland ecosystems such as has been seen with white-tailed deer (*Odocoileus virginianus*) in eastern North America (Pellerin, Huot & Côté, 2006), red deer (*Cervus elaphus*) in England (Welch & Scott, 1995), sika deer (*Cervus nippon*) in Japan (Takatsuki, 2009), and introduced sika deer in England (Hannaford, Pinn & Diaz, 2006). For instance, population growth of introduced sika deer in the Arne Saltmarsh, England, has decreased plant biomass and altered plant species composition ultimately resulting in the degradation of redshank (*Tringa tetanus*) habitat (Hannaford, Pinn & Diaz, 2006). Furthering our understanding of cervid ecology in wetland ecosystems is vital to conservation of these ecosystems.

In many ungulates, seasonal migratory and non-migratory individuals coexist within the same population. This phenomenon is known as “partial migration” (e.g., Hebblewhite & Merrill, 2007; Bolger et al., 2008; Singh et al., 2012; White et al., 2014). While migration imposes an energy cost on individuals (Bolger et al., 2008; Chapman et al., 2011), there are also numerous benefits such as avoiding predation, gaining access to nutritious food resources, and reducing competition among individuals (Fryxell & Sinclair, 1988; Hebblewhite & Merrill, 2007; Hebblewhite, Merrill & McDermid, 2008; Mysterud et al., 2011; Bischof et al., 2012; White et al., 2014). Therefore, whether an individual migrates or not directly affects its fitness and ultimately the population (Hebblewhite & Merrill, 2011; White et al., 2014; Rolandsen et al., 2016). Ungulate migration can lead to spatiotemporal variation in population density (Nelson, 1998; Mysterud et al., 2011), and spatial variation in ungulate distribution creates spatial heterogeneity in the ecosystem through changes in plant diversity and composition, predator behavior, and nutrient cycling (via excreta and carcasses) (Bump, Peterson & Vucetich, 2009; Hurley et al., 2012; Murray, Webster & Bump, 2013). The variation of timing of ungulate browsing in plant phenology determines how plants respond to the browsing (Hester et al., 2006; Takafumi et al., 2015). Therefore, understanding how many individuals in a population migrate as well as the migration start and end points are valuable information not only for ungulate conservation and management (Bolger et al., 2008; Singh & Milner-Gulland, 2011; White et al., 2014), but also for better understanding the ecosystem that they inhabit.

Sika deer (*Cervus nippon yesoensis*; from here on ‘deer’) is the only ungulate species inhabiting Hokkaido, the northern island of Japan. Before the Japanese extensively settled the island in the Meiji period (beginning in 1868) the deer were widely distributed across Hokkaido (Tawara, 1979). Owing to overhunting in the late 1800s and heavy snow in 1879, the populations size declined, and the distribution became limited (Kaji et al., 2010). After recovering from the population bottleneck in the 1950s, the population exploded, and its distribution re-expanded (Kaji et al., 2010). The reasons for population growth have been attributed to the extinction of the gray wolf *Canis lupus* by 1890, hunting regulation, replacement of native mixed-hardwood forests with conifer plantations, and increased pasture land (Kaji et al., 2010). This population growth is having a serious impact on farmland and the natural vegetation, especially in eastern Hokkaido (Kaji et al., 2000).

Currently, approximately 86% of all wetlands in Japan can be found in Hokkaido, and the majority of wetland areas in Hokkaido are located in the eastern part of the island (Kobayashi, 2016). The Kushiro Wetland, located in eastern Hokkaido, is the largest wetland in Japan and provides habitat for many endangered species including 73 endangered plants and vertebrates, such as the Red-crowned Crane (*Grus japonensis*) and the Japanese Huchen (*Hucho perryi*) (Ministry of the Environment, 2005). Kushiro Wetland is recognized as a valuable ecosystem and was listed as a Ramsar site in 1980. The main part of the wetland has been designated the Kushiro-shitsugen National Park and Wildlife Protection Area. Japanese law prohibits harvesting wildlife in the area, but deer hunting and pest control are permitted in surrounding areas. Previous studies have investigated deer population growth and its impact on the wetland. An aerial survey during winter showed that the deer population had increased by approximately 2.5–2.9 times from 1994 to 2015 (Ministry of the Environment, 2017). Deer trails detected from aerial photographs in the southern part of the wetland increased by 2.4 times in fens from 1977 to 2004 (Fujita et al., 2012) and by 4–8 times in bogs in from 2004 to 2010 (Muramatsu & Fujita, 2015). In comparison, deer trails also increased, by 1.9–2.6 times in the northern part of the wetland from 2004 to 2010 (Ministry of Environment, 2013). Deer browsing, trampling, and mud bathing has disturbed the primary vegetation, resulting in a shift to bare ground or novel plant communities (Fujita et al., 2012; Muramatsu & Fujita, 2015). For example, the abundance of bryophytes and dwarf shrubs decreased with changing microtopography, whereas annual plants (*Eriocaulon spp.*) increased in bogs (Muramatsu & Fujita, 2015). In comparison, in the fens, there was a change in dominant species from *Phragmites australis* and *Carex lyngbyei* to *Persicaria hydropiper* (Fujita et al., 2012).

On the basis of these circumstances, the Japanese Ministry of the Environment planned an ecosystem maintenance and recovery project to restore the Kushiro Wetland ecosystem to its pre-Ramsar Site registration state, i.e., how it was in or before 1980, which was scarcely affected by deer, by reducing the impact of deer on the wetland ecosystem. Previous studies have reported the impacts of deer on vegetation in the Kushiro Wetland (Fujita et al., 2012; Muramatsu & Fujita, 2015; Inatomi et al., in press), and the deer distribution was surveyed only during the winter (Inatomi, Uno & Ueno, 2014; Ministry of the Environment, 2017). However, deer seasonal movement patterns, which are essential information for achieving more effective deer management, have not been thoroughly studied. If deer migrate outside the wetland, then the population dynamics of the deer and harvest pressure by humans around the wetland could affect the interactions between the deer and the ecosystem within the wetland. On the other hand, if the deer migrate to other areas within the boundaries of the wetland, it is reasonable to assume that the spatial distribution of the impact on the ecosystem varies seasonally. Moreover, if deer inhabiting the wetland consist of multiple seasonal movement patterns, ecosystem managers must consider adapting their management strategies to correspond to each movement pattern.

This study aimed to clarify the movement patterns and area use of the Kushiro Wetland by deer. To this end, GPS location data for the deer were used to classify individual seasonal movement patterns, to estimate the degree of utilization of the wetland by individuals, and to calculate the extent to which home range area overlapped among capture sites. On the basis of these results, we discuss factors related to deer use patterns of the Kushiro Wetland and the implications for ecosystem management and deer management in the wetland.

## Methods and Materials

### Study area

The Kushiro Wetland (20,366 ha) is Japan’s largest wetland, most of which makes up Kushiro-shitsugen National Park (Ministry of the Environment, 2005). The center of the park has been designated a Wildlife Protection Area and a Ramsar site. Kushiro Wetland is comprised of various vegetation types. The fen area of the wetland is dominated by *Phragmites australis* and *Carex* spp., and the wetland forests feature mainly *Alnus japonica*. Bogs comprise the smallest part of the wetland and mainly consist of *Sphagnum* spp. Annual average temperature and precipitation between 1981 and 2010 were 5.5 °C and 1,119.6 mm, respectively, and monthly mean maximum snow depth per day in February was 25.9 cm between 1985 and 2016 (at the Tsurui Weather Station, which is close to the study area; obtained from the Japan Meteorological Agency, http://www.data.jma.go.jp/obd/stats/etrn/, 2017/1/15), which is shallow compared to other regions in Hokkaido.

### Deer location data

**Figure 1:**
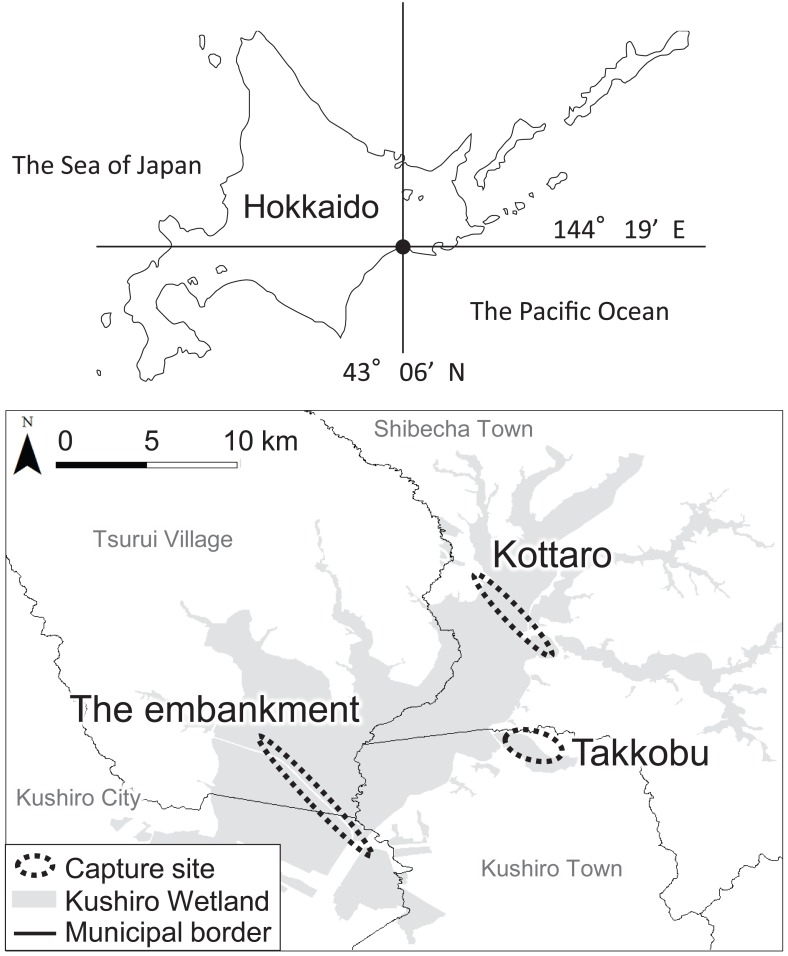
Locations (*n* = 3) where tracked sika deer (*Cervus nipponyesoensis*; *n* = 28) were captured in Kushiro Wetland. The boundary of the wetland was obtained from the Kushiro Wetland Restoration Project Shitsugen Data Center (http://kushiro.env.gr.jp/index.html).

A total of 28 hinds, 27 adults (i.e., over the age of three) and one yearling, were captured in three different designated areas (capture sites) inside Kushiro Wetland (Fig. 1) and fitted with GPS collars (IridiumTrackM2D, LOTEK). The deer were still-hunted with tranquilizer guns, without any bait. The data from one of the capture sites (Lake Takkobu) formed part of the “Capturing method evaluation of deer in Kushiro-shitsugen National Park in 2014” project of the Ministry of Environment. Permission to capture and handle wildlife including animal welfare and ethics, was obtained from the Hokkaido government (Approval Number: 176-5 and 423-5), and permission to capture and handle wildlife in a wildlife protection area, including animal welfare and ethics, was obtained from The Ministry of Environment (Approval Number: 1409291 and 1510071). Permission for the field study on government land was obtained from the Hokkaido Development Bureau (Approval Numbers: 68, 69 and 105), complying with the current laws and regulations of Japan.

Three capture sites were selected: one in the north, one in the center, and one in the south of the wetland, all in accessible areas. We focused only on hinds, as they are key factors driving population dynamics in polygynous ungulates (Gaillard et al., 2000). The first capture site was located north of Lake Takkobu (from here on ‘Takkobu’). The second site was at the Right Embankment of Kushiro Wetland, which runs through the southwestern section of the wetland (from here on the ‘embankment’). The third and last site was located in the northern part of the wetland near Prefectural Route 1060, which runs across the northern part of the wetland from Kottaro Observatory to National Route 391 (from here on ‘Kottaro’). GPS collar fitting was carried out in February 2014 (one collared hind) and February–March 2015 (seven collared hinds) at Takkobu. Ten more hinds were fitted with collars at the embankment October–November 2014 and another ten were fitted at Kottaro in February 2015. Collar data were obtained at a fixed interval of every 3 h. The fix rate for all individuals was 99.93%. Three individuals whose location datasets did not span a full year were omitted from all data analyses. Two of these individuals moved out of the wetland and were harvested by humans in the area, and the signal from the third individual’s GPS collar was lost after it traveled 60 km away from the wetland into the nearby Japan Ground Self-Defense Forces base in Betsukai town.

### Classification of seasonal movement patterns

To quantitatively classify seasonal movement patterns using the net squared displacement (NSD) method (Bunnefeld et al., 2011), described below in detail, the datasets were required to include 365 time steps (i.e., one-year of data) from the start day (Bunnefeld et al., 2011); thus, we used one-year of location data for each individual in all analyses. The data collecting periods encompassed the time from when the collars were set to when their drop-off mechanisms were activated. The start days for deer in Takkobu were February 14, 2014 (*n* = 1), February 14, 2015 (*n* = 3), March 17, 2015 (*n* = 1), and March 18, 2015 (*n* = 1). For all remaining individuals, the start day was March 1, 2015. One point/day (at noon) was selected from the movement trajectories for seasonal movement pattern classification, and all location data were used for the other analyses.

Following the recommendations of Cagnacci et al. (2016), we used a combination of results of two methods to classify the seasonal movement patterns of deer: analysis of NSD (NSD method) and analysis of overlapping individual winter and summer home ranges (overlap method). Utilizing this combination enabled us to discriminate between ‘true’ seasonal migrants and individuals that only make minor seasonal movements from their home range (Cagnacci et al., 2016).

**Figure 2:**
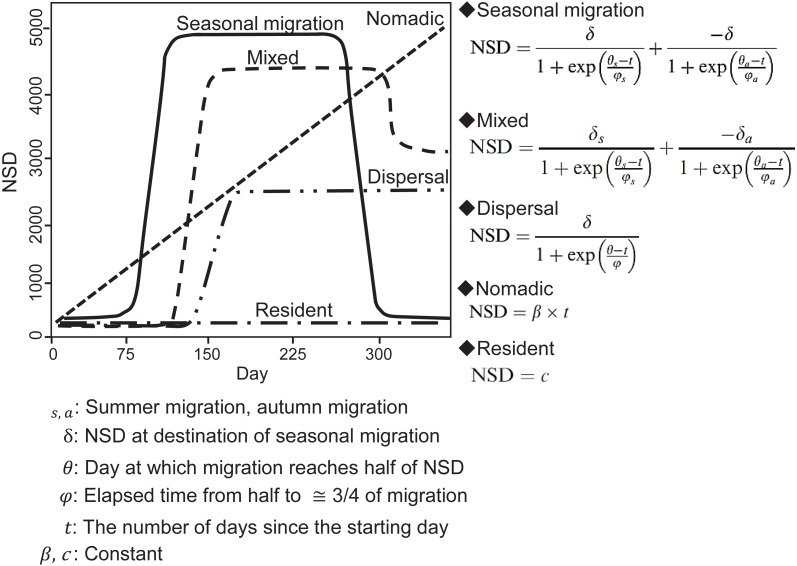
Models of seasonal movement patterns based on the net squared displacement (NSD) method and expected NSD plots for different seasonal movement patterns. Solid line, seasonal migration; long dashed line, mixed; two dot-and-dash line, dispersal; short dashed line, nomadic; dot-and-dash line, resident. Model functions and plots are modified from Bunnefeld et al. (2011).

In an NSD analysis, the squared distance between each location and the location where an individual was on the start day is calculated and indicates how far an individual is on a given day from where it was on the start day. By fitting different theoretical seasonal movement models based on the pattern of the NSD time series (Fig. 2), such as seasonal migration and dispersal, the NSD method quantitatively classifies individual seasonal movement patterns and estimates the timing of migration initiation and duration of time spent in the seasonal home range for seasonal migration individuals (Bunnefeld et al., 2011). To identify seasonal movement patterns using the NSD method, we calculated NSD values for the location at noon every day for each individual using the adehabitatLT package (ver. 0.3.21) (Calenge, 2006) in R (ver. 3.2.4, R development Core Team, 2016). By selecting the best fit theoretical movement models, the results were classified according to Bunnefeld et al. (2011) into seasonal migration, mixed (seasonal movement away from a home range, as with seasonal migration, returning to inexactly the location of departure, but to a nearby area), dispersal (seasonal movement away from a home range, as with seasonal migration, but settling in a home range in a new area), nomadic (random movements), and resident (lacking long distance movement, no difference in home range area between seasons). Following the definition of ‘seasonal’ by Cagnacci et al. (2016), individuals had to remain more than 30 days at a location to avoid misclassifying short visitors and migrants, who remain in seasonal home ranges continuously. If a statistically best fitted model had less than 30-days duration in a given season, the second best fitted model was selected. Model selection was based on the concordance criterion (Börger & Fryxell, 2012), and evaluation was performed using the nls.lm function of the minipack.lm package (ver 1.2-0) in R.

Next, the overlap method was applied for to all individuals. The overlap method distinguishes among seasonal movement patterns, classifying them as seasonal migration, resident, no-return (seasonal movement away from a home range, as with seasonal migration, but without returning to the home range of the preceding year) by evaluating whether the degree of overlap of the home range before and after seasonal movement is smaller than the threshold values (Cagnacci et al., 2016). We used seasonal time intervals to compute home range overlap among seasons according to Cagnacci et al. (2016). The period of each season was determined by shifting time windows (resolution of one month), with all possible combinations of two- or three-seasonal ranges (first winter–summer–second winter). Home range overlaps were estimated for each period using “kernelUD” function with bivariate normal kernel in the adehabitatHR package (ver. 0.4.14) (Calenge, 2006). The degree of home range overlap was evaluated using Bhattacharyya’s affinity index (Bhattachayya, 1943). Movement patterns were classified according to Cagnacci et al. (2016). If the minimum seasonal overlap of an individual is above 15% (threshold), the individual was classified as “resident”. If the minimum overlap is below the threshold, the individual was further distinguished between seasonal migration and “no-return” (including dispersal or nomadic; however, the overlap method cannot distinguish dispersal and nomadic). If the overlap between the first winter and second winter of the individual exceeds 50%, the individual was classified as “migratory;” if not, it was classified as “no-return.” An individual was also classified as a no-return when its minimum overlap occurred at a combination of time windows, with only two seasonal ranges.

We finally classified all individuals by combining the results of the classification from the NSD method and Overlap method (Table 1). Trajectories for which the classification results of the NSD method and Overlap method did not concur were classified as atypical (short and/or multiple trips between home ranges) (Cagnacci et al., 2016). Finally, the movement patterns for all individuals were classified as seasonal migration, mixed, dispersal, nomadic, resident, and atypical. Because no individuals were classified as mixed and nomadic in the final classification, a description of the seasonal movement types for the other analyses, e.g., estimating home range overlap with the wetland, were abbreviated in the following methods sections.

**Table 1:** Protocol of combining results from the NSD method and Overlap method to determine final classification for the seasonal movement type of individuals.

Classification by the NSD method	Classification by the Overlap method	Final classification
Seasonal migration	Seasonal migration	Seasonal migration
Mixed	No-return	Mixed
Dispersal	No-return	Dispersal
Nomadic	No-return	Nomadic
Resident	Resident	Resident
Seasonal migration	No-return or Resident	Atypical
Mixed	Seasonal migration or Resident	Atypical
Dispersal	Seasonal migration or Resident	Atypical
Nomadic	Seasonal migration or Resident	Atypical
Resident	Seasonal migration or No-return	Atypical

To investigate where the home range centers of each individual were during the summer and both winter periods and compare to previous studies about sika deer, we calculated the centers of activity (COAs) by averaging the locations for each deer in every season (Hayne, 1949), as has been done in previous studies of sika deer (e.g., Igota et al., 2004). We mapped the results of COAs. Each COA period was defined by applying the estimated parameters calculated with the NSD method according to seasonal movement patterns. For migrant individuals, the estimated period for each individual was used directly. For two migrants (ID = 36,725 and 36,728), their last location points during the tracking period were defined as the COA of the second winter. This definition was used because the NSD method estimated that they had still had not reached the second winter habitat on the last day of the period, whereas the overlap method classified them as seasonal migration (not “no-return”, namely, they had returned to previous winter home range). The individuals with movement types other than seasonal migration did not perfectly move with the seasons, and their periods of seasons were not uniquely defined (Fig. 2). Therefore, to compare the location of deer in the same seasons (first winter, summer, second winter) among seasonal movement types and capture sites, for the other migrants, all or a part of the periods were defined based on the periods of migrants. This period was defined as all migrant individuals remained in their seasonal home ranges (for the second winter, excluding two irregular migrants, which were estimated as they still have not reached the second winter habitat by the NSD method). For dispersing individuals, the estimated period for each individual during the first winter, which is the season occurring before dispersal, was determined by the NSD analysis. The summer and second winter periods were defined as the periods in which all migrant individuals remained in their summer and second winter home ranges. For resident and atypical individuals, the periods in which all migrant individuals remained in their winter and summer home ranges were used to define the periods.

### Seasonal migration timing and distance

Snow depth has been reported to affect the migration behavior of cervids (Mysterud et al., 2011), and the timing of migration initiation has been shown to be related to the timing of snow melt in eastern Hokkaido (Uno & Kaji, 2000). To examine the relationship between snow depth and the timing of migration initiation, we compared the state of snow accumulation and migration initiation. The date of loss of snow cover (defined as the first day snow depth fell below 1 cm) and the date of first snow cover (defined as the first day snow accumulation exceeded 1 cm) were obtained from the Japan Meteorological Agency data for the Tsurui Meteorological Weather Station (N43°14′, E144°20′) near the study area. We compared the date of loss of snow cover and the date of first snow cover with the timing of migration initiation for spring and autumn, respectively, which were estimated by the NSD method.

Additionally, for the sake of comparing migration distance determined in this study with that of previous studies, the distances between summer and winter COAs were calculated for each migrant individual.

### Degree of wetland utilization and home range overlap among capture sites

To estimate the degree of wetland utilization of each individual as one of the indicators of their pressure on the wetland, the wetland area per annual home range for each individual was calculated by dividing the total area of the wetland in a given home range by the annual home range area. The annual home ranges were estimated using the adehabitatHR package in R and mapped by creating a 95% local convex hull (LoCoH) (Getz & Wilmers, 2004; Getz et al., 2007).

To evaluate the difference in the overlap of the utilization distribution of deer among intra- and inter-capture sites, the amount of the overlap of home ranges for each individual among the intra- and inter-capture sites were calculated both annually and for each season. The overlap of annual home ranges among individuals was calculated by using the annual home range of each individual, which was also used to estimate wetland utilization. To calculate the overlap of seasonal home ranges among individuals, seasonal home ranges were estimated each individual with same duration of seasons for estimation of COA. The overlap area of the seasonal home ranges was calculated for each individual among the intra- and inter-capture sites. The seasonal home ranges was used to evaluate the constant utilization distribution during a season because it excludes seasonal movement periods. The ratio of the area of overlap of a given home range of an individual with other individuals in the intra- and inter- capture sites to the total home range area of the individual was calculated annually and seasonally, i.e., for the first winter, summer, and second winter. The overlapping areas were averaged per capture site. The overlap of the second winter home ranges of two migrants was not estimated as they had still been moving to the second winter habitat at the last location when using the NSD method, because it was difficult to define the duration of the second winter.

Estimations for home ranges and statistical analyses were performed with R (ver. 3.2.4, R development Core Team, 2016). Home range sizes, the area of overlap of home ranges and wetland area, and the area of home range overlap among the intra- and inter-capture sites of individuals were calculated with ArcGIS (ver. 10.3.1).

## Results

**Figure 3:**
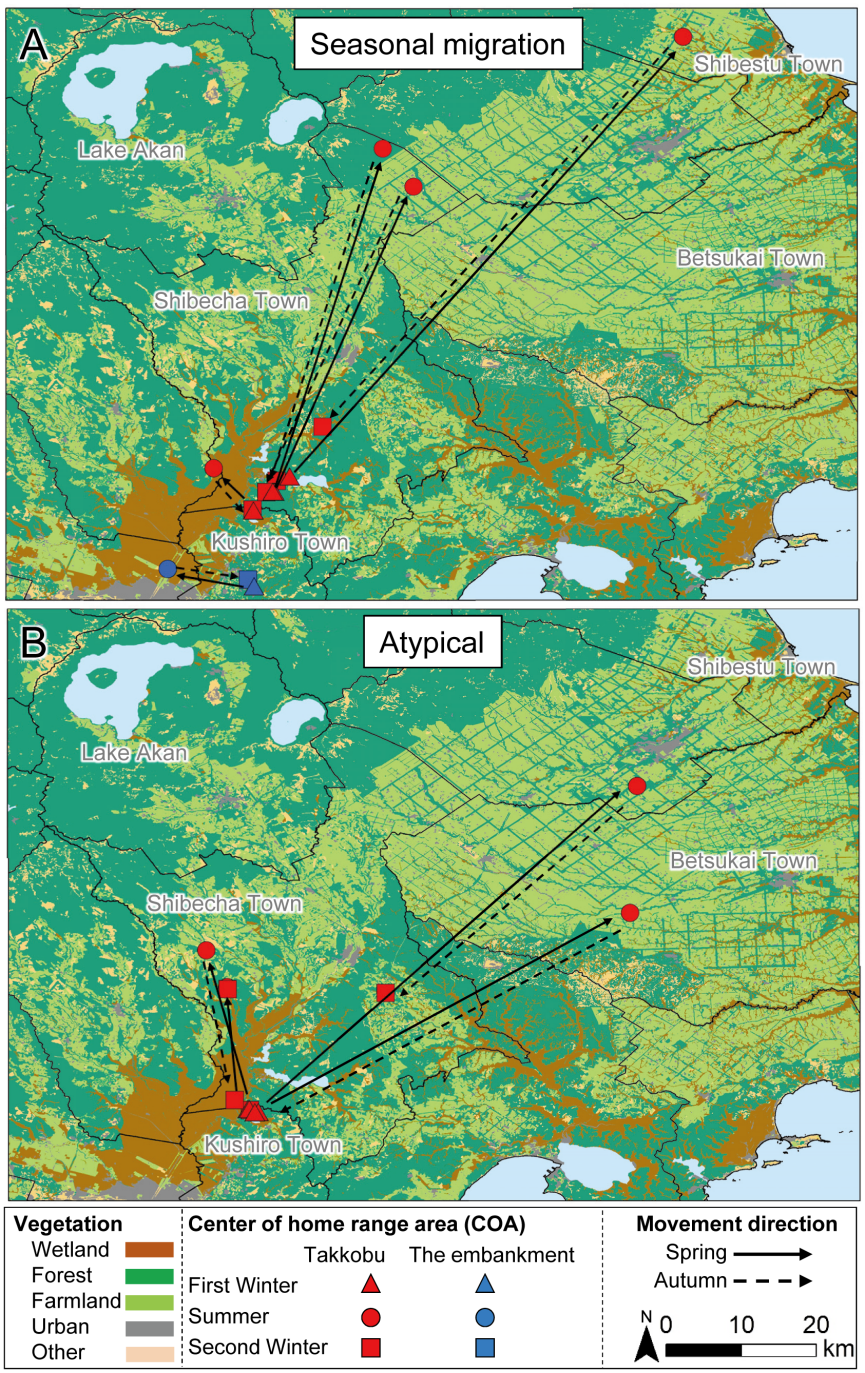
Movement patterns of sika deer (*Cervus nippon yesoensis*) with seasonal centers of activity (COAs) occurring outside Kushiro Wetland at some pointduring the year. Movements of (A) seasonal migration and (B) atypical individuals are shown. Dots indicate COAs for winter and summer for each individual. Lines connect individual COAs, whereas arrows show the direction of movement in spring and fall. All deer captured at Takkobu (*n* = 8) and one deer captured at the embankment are shown. Deer captured at Kottaro are not shown because their COAs were entirely within the wetland.

**Figure 4:**
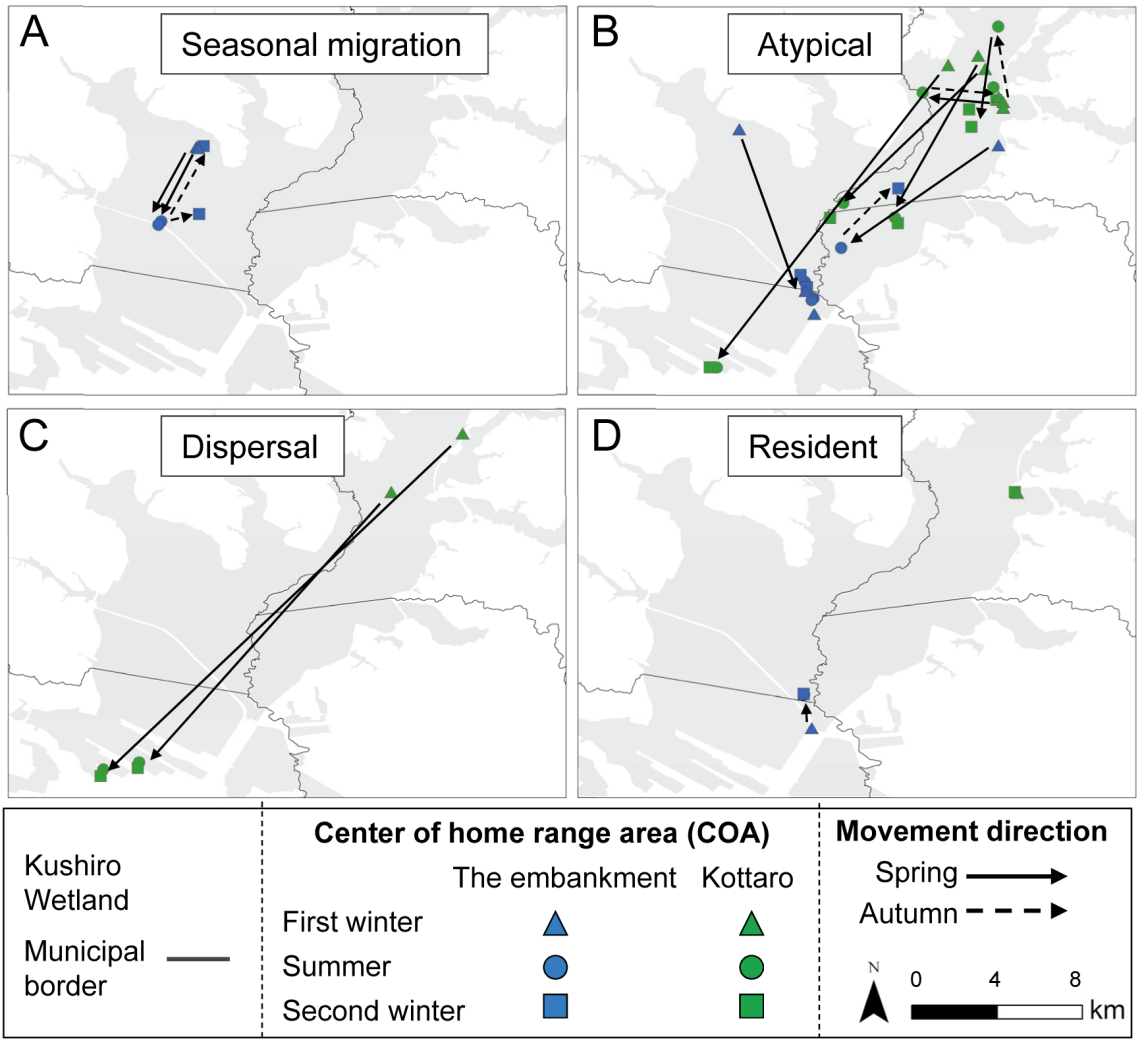
Movement patterns of sika deer (*Cervus nippon yesoensis*) that remained within the wetland all year. (A) Seasonal migration, (B) Atypical, (C) Dispersal, (D) Resident. Sika deer captured at the embankment (*n* = 7) and Kottaro (*n* = 9) sites are shown, but deer captured at Takkobu are not because all Takkobu individuals had at least one seasonal center of activity (COA) outside the wetland. Dots indicate COAs for winter and summer for each individual. Lines connect individual COAs, and arrows show spring or autumn movement direction, except for those of residents.

All capture sites contained multiple individuals with differing movement patterns, with no individuals being classified as mixed or nomadic (Table 2). A total 16 individuals were classified as atypical, with the classified movement type differing between the NSD method and Overlap method (Table S1). Half of the individuals from Takkobu were classified as migrant individuals along with three deer from the embankment. Takkobu migrants spread over a large area to agricultural areas in the towns of Shibetsu, Betsukai, and Shibecha, as well as to Tsurui Village (Fig. 3). One individual from the embankment migrated to an urban area in the town of Kushiro and to a forested area close to a quarry. Another migrant from the embankment had its COAs in different areas within the wetland. Regarding the atypical movements of Takkobu individuals, two individuals did not return from their wintering ranges with one moving 16 km northwest of the wetland and establishing a home range outside the wetland during the second winter. Approximately half of all individuals had COAs within the wetland year-round regardless of their movement patterns (Fig. 4).

**Table 2:** Seasonal movement pattern classifications per sika deer (*Cervus nippon yesoensis*) capture site.

Capture site	Seasonal migration	Dispersal	Resident	Atypical
Takkobu (*n* = 8)	4	0	0	4
The enbankment (*n* = 8)	3	0	1	4
Kottaro (*n* = 9)	0	2	1	6

**Notes.**

One of the seasonal migrant individuals from Takkobu was tracked from February 2014 to February 2015. The remaining individuals were tracked from March 2015 to March 2016.

There was no clear relationship between the timing of migration initiation and the first day of snow cover and melted snow cover, even though individuals captured in Takkobu during 2016 initiated spring migration four days after the loss of snow cover (Table S2). Greater variation among individuals was detected for the autumn migration compared to the spring migration. Most individuals exhibited substantial variation in when they started the autumn migration, with as much as a one-month difference between individuals.

Individuals from Takkobu migrated up to four times farther than embankment individuals did. Average spring migration distances were 26.2 ± 9.3 km (mean ± SE) (range 4.0–69.9 km) for all migrants, 41.6 ± 11.3 km for Takkobu migrants, and 5.7 ± 1.3 km for embankment migrants. Average autumn migration distances were 24.5 ± 9.5 km (range 1.5–62.6 km) for all migrants, 39.4 ± 10.2 km for Takkobu migrants, and 4.6 ± 1.9 km for embankment migrants.

Mean annual home range sizes was 6.8 km^2^ ± 1.8 (SE) (Takkobu: 16.5 km^2^ ± 3.6, the embankment: 2.4 km^2^ ± 0.2, Kottaro: 2.1 km^2^ ± 0.3) (Table 3). The degree of wetland utilization differed substantially among individuals and ranged from 2.7 to 100.0% (Table 3). Eleven individuals had home ranges that consisted largely of wetland with two of these deer using the wetland exclusively (i.e., 100% utilization), whereas 81%–96% of the home range of the other nine deer encompassed the wetland. The degree of wetland utilization did not tend to differ among movement patterns. However, Takkobu individuals tend to use the wetland less than deer from the other capture sites (Takkobu: 21.2%, the embankment: 76.8%, and Kottaro: 75.9%).

**Table 3:** Annual home range size, amount of home range overlapping the wetland, and wetland utilization (percentage of home range overlapping wetland area). Wetland utilization was calculated by dividing the amount of wetland in a home range by the annual home range size.

Capture site	Movement type	Annual home range size (km^2^) ± SE	Amount of wetland in annual home range area (km^2^) ± SE	Wetland utilization (%) ± SE	Range of wetland utilization (%)	*n*
Takkobu (*n* = 8)	Migration	21.1 ± 5.5	3.2 ± 0.8	20.2 ± 5.6	9.9–39.0	4
	Atypical	12.2 ± 3.6	2.0 ± 0.7	22.2 ± 6.7	2.7–40.0	4
	Mean	16.7 ± 3.6	2.6 ± 0.6	21.2 ± 4.4	2.7–40.0	
The embankment (*n* = 8)	Migration	2.8 ± 0.1	1.9 ± 0.7	67.4 ± 23.5	9.9–96.4	3
	Resident	2.5	2.0	81.3	–	1
	Atypical	2.1 ± 0.3	1.8 ± 0.3	82.7 ± 5.6	64.9–93.6	4
	Mean	2.4 ± 0.2	1.8 ± 0.3	76.8 ± 9.6	9.9–96.4	
Kottaro (*n* = 9)	Dispersal	2.6 ± 0.0	1.4 ± 0.3	53.7 ± 11.4	37.6–69.9	2
	Resident	0.5	0.3	60.4	–	1
	Atypical	2.2 ± 0.3	1.8 ± 0.2	85.8 ± 6.7	52.1–100.0	6
	Mean	2.1 ± 0.3	1.5 ± 0.2	75.9 ± 7.0	37.6–100.0	
All		6.8 ± 1.8	2.0 ± 0.2	58.7 ± 6.6	2.7–100.0	25

**Notes.**

SE, standard error

**Figure 5:**
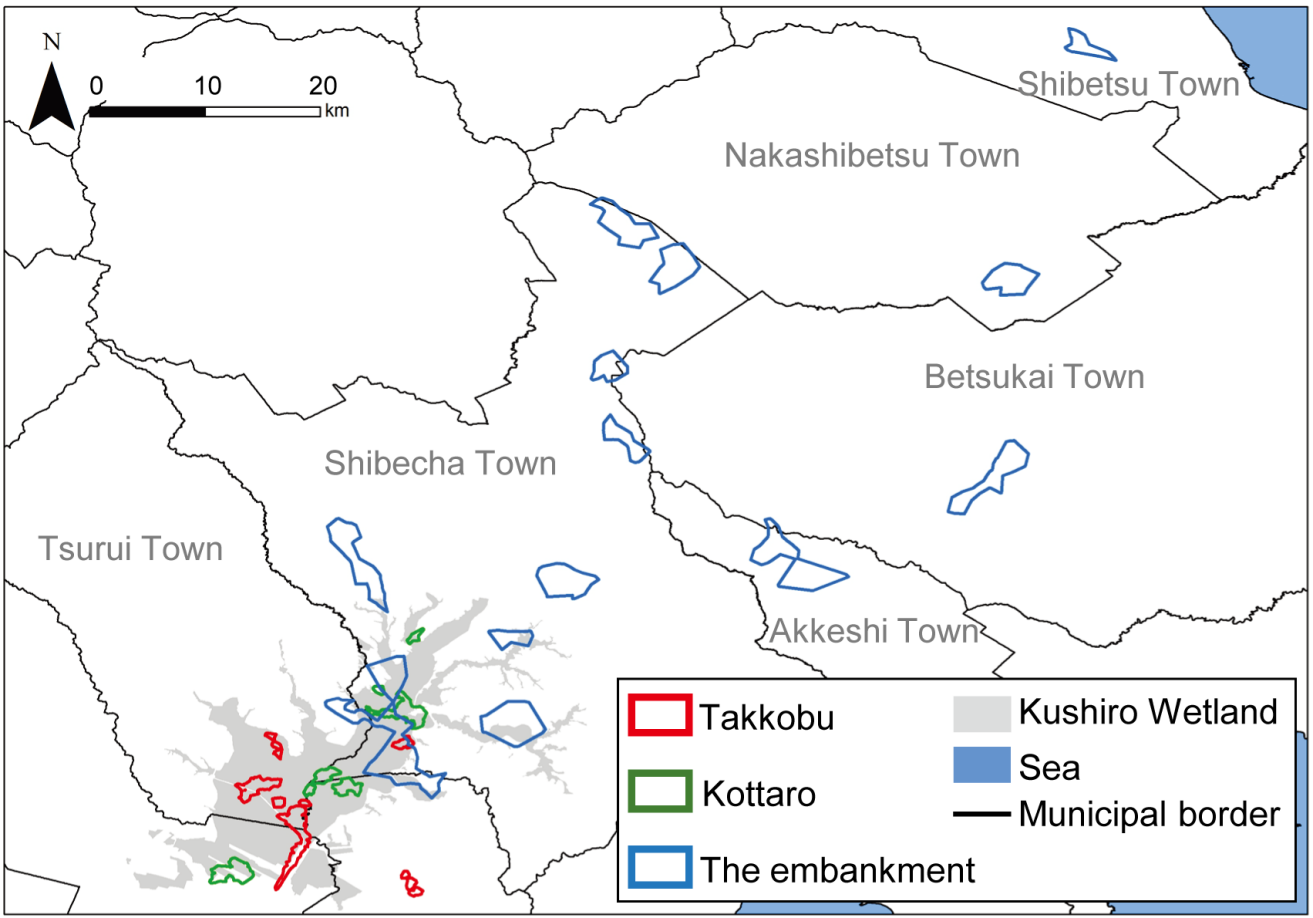
Annual home ranges of sika deer (*Cervus nippon yesoensis*, *n* = 25) in the Kushiro Wetland. Home ranges were estimated by a 95% local convex hull by all recorded locations of a deer. Red outline, home ranges of individuals captured at Takkobu; green outline, home ranges of individuals captured at Kottaro; blue outline, home ranges of individuals captured at embankment.

The overlapping areas of the annual and seasonal home range of individuals among intra-capture sites were larger than individuals among inter-capture sites (e.g., the mean of the annual home range overlapped with intra-capture sites by 47.7% and inter-capture site by 1.3%) (Table S3). The annual individual home range overlapped slightly with inter-capture sites (Fig. 5, Table S3); specifically, 0.5% of Takkobu home ranges (0.5% with the embankment, 0.0% with Kottaro), 3.0% of embankment home ranges (3.0% with Takkobu and 0.0% with the Kottaro), and 0.3% of Kottaro home ranges (0.0% overlap with embankment and 0.3% overlap with Takkobu) overlapped with those of other home ranges. There were very few (0.0–3.4%) overlapping seasonal home ranges among the capture sites for any season (Fig. S1, Table S3).

## Discussion

Individuals from all capture sites exhibited a variety of movement patterns, e.g., resident and seasonal migration, even though they inhabited the same area at one point during the year. These results indicate that the sika deer population in Kushiro Wetland is a partially migratory. Partially migratory ungulate populations consist of individuals that exhibit variation of seasonal movement behavior in the residency-to-migration continuum among individuals (Ball, Nordengren & Wallin, 2001; Dingle & Drake, 2007). In general, when the seasonality of resources, predictability of seasonality, and density-dependent competition are high, the ratio of migrants in the population is high; in the opposite scenario, the ratio of residents in the population is high (Jonzén et al., 2011; Mysterud et al., 2011; Eggeman et al., 2016). Therefore, if such environmental and intra-population conditions are moderate, the ratio of individuals that exhibit intermediate movement behavior in the residency-to-migration continuum (i.e., “atypical” individuals) is high in the population. Cagnacci et al. (2016) applied both the NSD method and Overlap method to roe deer (*Capreolus capreolus*), red deer, and reindeer (*Rangifer tarandus*) populations. The authors found that the results obtained from NSD method and Overlap method were approximately 50–90% consistent, with atypical movement individuals representing approximately 10–50% of the population. Our study showed that 56% of individuals in the population in the Kushiro Wetland were atypical. These results were not significantly out of the range, but were relatively high compared to the range. Thus, the population of the Kushiro Wetland was likely to be under more moderate seasonality or predictability of resource availability or density-dependent competition than the populations in the previous study.

A total of 28% of all individuals exhibited a high degree of wetland utilization meaning that they inhabited the wetland exclusively or to a large extent year-round. The individuals may inhabit the wetland to avoid hunting risk (Lone et al., 2015) as the majority of this area is a Wildlife Protection Area, which could allow these individuals to continue to increase until reaching the carrying capacity of the wetland. After the extinction of the gray wolf around 1890 (Inukai, 1933) in Hokkaido, the main cause of deer mortality in eastern Hokkaido has primarily been harvest by hunters with adult female mortality rates estimated at 0.118 (harvest) and 0.053 (natural) (Uno & Kaji, 2006). Furthermore, movements of some Takkobu individuals that exhibited atypical movements, such as leaving the wetland for surrounding areas and establishing new home ranges there, indicated that Kushiro Wetland may be a population source in eastern Hokkaido.

Our results showed that seven deer seasonally migrated into the wetland. Previous studies have highlighted factors for seasonal migration, such as predation risk avoidance (Hebblewhite & Merrill, 2007; White et al., 2014), access to nutritional resources (Fryxell & Sinclair, 1988; Hebblewhite, Merrill & McDermid, 2008; Bischof et al., 2012), adverse weather effects (Fryxell & Sinclair, 1988; Mysterud et al., 2011), and social interaction avoidance due to density (Mysterud et al., 2011). The predation risk avoidance and nutritional resources hypotheses may be supported by the migration data observed in the present study; however, we could not examine the avoidance of social interactions hypothesis and adverse weather effects since accurate information about summer deer density and weather information at sufficiently fine spatial scale are limited. Takkobu migrant individuals moved into the Kushiro Wetland in winter, and they moved out of the wetland in summer. Sport hunting and pest control are conducted outside of the wetland with the open season spanning from October to March, and pest control is conducted year-round. However, harvests are prohibited in the wetland and deer, therefore, can reduce mortality risk here, especially during the open season. In fact, hunters harvested two individuals moving out of the wetland during winter, but no tracked deer died in the wetland. The summer habitats of Takkobu migrant individuals were agricultural areas in places such as Shibecha Town. These individuals can browse highly nutritional crops grown in the summer. The movement patterns of Takkobu migrant individuals suggested that they move into the wetland to avoid predation risk in the winter and out of the wetland into agricultural areas in summer because the benefit of foraging on these high nutritional resources exceeds the predation risk. On the other hand, one of the migrant individuals from the embankment moved out of the wetland in winter and back into the wetland in summer. The winter habitat of this individual was forested land near urban areas and developed land where hunting pressure is thought to be slightly lower due to legal constraints imposed by the Japanese Firearms and Swords Control Law. In summer, many embankment deer have been observed feeding on pasture grasses planted on the slopes of the embankment to prevent erosion (Ministry of the Environment, 2017). Pasture grasses have higher nutritional value compared to naturally growing plants, e.g., *Phragmites australis*, and are often foraged by sika deer (Takatsuki, 2001; Tsukada, Fukasawa & Kosako, 2008). It seems, therefore, that migrant deer move to the embankment during summer to access nutritional food resources. Another migrant individual migrated from the embankment to another area of the wetland in winter, i.e., it migrated within the wetland. This individual may have used the embankment in the summer for the sake of accessing high nutritional food resources, but the reasons for the individuals leaving the embankment in winter is uncertain.

The results of the present study showed that the proportions of movement patterns differed among individuals from different capture sites, even though the capture sites themselves were geographically near one another. The factors for determining the proportion of migrants in an ungulate population have been debated (Bolger et al., 2008; Chapman et al., 2011). Taking into account the environmental factors, deer survival rates, and nutritional status of individual deer (White et al., 2014) in future studies of Kushiro Wetland’s deer population would contribute to identifying the factors determining the proportion of the migrants in the population.

Snow depth is one of main factors that influences the migration behavior of cervids (Mysterud et al., 2011). A study conducted in Akan, located approximately 40 km northwest of Kushiro Wetland, found that snow cover minimally affected autumn migration, whereas, spring migration started soon after the timing of snow melt in May (Uno & Kaji, 2000). However, in the present study, no clear relationship was found between snow cover and migration initiation, except for individuals captured in 2015 in Takkobu. This could possibly be due to the short snow cover period and shallow snow depth in Kushiro Wetland compared to Akan. Uno & Kaji (2000) reported a total of 121 days with a snow depth over 50 cm during the study period (1993–1996) with snow melting in mid-May. On the other hand, in Kushiro Wetland, snow depth was only 20 cm in February 2015 (Ministry of the Environment, 2016), and the snow melted in early April. Factors besides snow cover, e.g., plant phenology (Albon & Langvatn, 1992; Rivrud et al., 2016) might also influence the timing of sika deer migration in the Kushiro Wetland, and should be assessed in future studies.

In the present study, the average migration distance during spring for deer was 26.2 ± 9.3 km, and it was 24.5 ± 9.5 km during autumn. These distances were similar to those observed for deer in Shiranuka located approximately 50 km west of Kushiro Wetland (35.1 km) (Igota et al., 2004) and in Akan (19.9 km, spring migration; 24.3 km, autumn migration) (Uno & Kaji, 2000), but longer than those for deer in Okuchichibu (15.9 km) (Takii et al., 2012) and Kirigamine (9.9 km) (Takii, Izumiyama & Taguehi, 2012) on the main island of Japan, south of Hokkaido. This trend is in accordance with a previous study that indicated that moose (*Alces alces*) have longer migration distances at northern latitudes (Singh et al., 2012).

In addition, our results showed that the overlap of individual home ranges among intra-capture sites was higher than among inter-capture sites. Furthermore, the overlap of the home ranges among the three capture sites exhibited little to no overlap with the annual home ranges or in their seasonal home ranges. Thus, individuals from different capture sites appear to use discrete geographical ranges as habitats. The capture sites represent prospective sites for deer population control due to their accessibility and location. The results imply that the sites could be treated as separate management units.

## Conclusions & Management Implications

This study demonstrated that deer exhibit several types of seasonal movement behaviors in Kushiro Wetland. A quarter of the tracked deer used the wetland as their main habitat year around. Furthermore, large numbers of deer moved in and out of the wetland, and the degree of wetland utilization differed among capture sites and individuals. Therefore, ecosystem maintenance and recovery projects in Kushiro Wetland should consider the movement behavior and geographical utilization distribution of deer to effectively manage these animals in the wetland. If harvest is conducted in the wetland in winter, it should be implemented before migrants move out of the wetland. In addition, many seasonal migrant individuals possibly spend the winter in the wetland as a strategy to avoid hunting, and browse on highly nutritional crops in the surrounding agricultural areas during summer. Thus, to manage these individuals, both ecosystem management in the wetland as well as agricultural countermeasures in the surrounding areas need to be considered together. For example, to harvest more migrants in the summer home ranges, the hunting season should start earlier (Loe et al., 2016) around the wetland, while pest control should be encouraged in farmland. Furthermore, in terms of pasture grasses on the embankment, ecosystem managers should recognize that growing these grasses on the embankment is a conservation issue not only because they are exotic and planted in the core area of the wetland, but also because they would provide favorable habitats for migrant and resident deer.

Land-use development in and around Kushiro Wetland has caused marked vegetation modification due to sediments and nutrients being carried and deposited from upstream watersheds (Nakamura, Kameyama & Mizugaki, 2004) and is one of the major conservation issues of the wetland. Ungulates transfer nitrogen and phosphorous from farmlands to forests through their movement (Seagle, 2003; Abbas et al., 2012). In our study, deer migrated from farmland areas to the wetland; thus, the deer likely provided cross-ecosystem nutrient subsidies from the farms to the wetland through their excreta and carcasses in addition to cross-ecosystem browsing impacts on vegetation (e.g., Bergman & Bump, 2015; Takafumi et al., 2015). Although an overwhelming amount of nutrients flow from upstream watersheds to the wetland (Nakamura, Kameyama & Mizugaki, 2004), ungulate excreta and carcasses change the spatial distribution of soil nutrients, ultimately leading to changes in plant nutrient contents and plant community composition (Bump, Peterson & Vucetich, 2009; Murray, Webster & Bump, 2013). Therefore, both biological interactions, such as browsing, and biogeochemical ecosystem processes, such as subsidies from farmlands, should be considered when evaluating the impacts of deer on wetland ecosystems.

Deer were historically distributed across the entire island of Hokkaido (Tawara, 1979). In areas with native ungulate populations, moderate ungulate browsing leads to high plant diversity, and browsing occurring in a mosaic across the landscape would have promoted high plant diversity at a landscape scale through the spatial heterogeneity of the plant community affected by the ungulates (Royo et al., 2010). Our results suggested that the density of the deer population in the wetland is spatiotemporally variable, because ungulate migration is known to exhibit this type of variation (Nelson, 1998; Mysterud et al., 2011). Therefore, for the ecosystem maintenance and recovery project in Kushiro Wetland, not only is there a need to manage deer population size itself, but also to fully understand the interaction between the spatiotemporal variation of deer impacts and vegetation at the landscape level. The combination of more detailed information on the spatiotemporal distribution of deer density caused by the seasonal movements of individuals and different responses to deer browsing among vegetation types (Inatomi et al., in press) would provide essential information for ecosystem management at a landscape level in Kushiro Wetland.

##  Supplemental Information

10.7717/peerj.3869/supp-1 Figure S1 Seasonal home range area of sika deer (*Cervus nippon yesoensis*) in the Kushiro Wetland during (A) the first winter, (B) summer, and (C) the second winter in a year The home range area was estimated by all recorded locations of deer and determining the 95% local convex hull for them. The periods of the seasons were defined as well as the definition used in the COA calculation (see methods section) Red outline, home ranges of individuals captured at Takkobu; green outline, home ranges of individuals captured at Kottaro; blue outline, home ranges of individuals captured at embankment; shaded grey area, Kushiro Wetland; filled blue areas, sea; solid lines, municipality boundaries.

10.7717/peerj.3869/supp-2 Table S1 The classification results of the NSD method and Overlap method, and final classification results

10.7717/peerj.3869/supp-3 Table S2 Dates of spring and autumn migration initiation of sika deer (*Cervus nippon yesoensis*) classified as seasonal migrants in 2014 and 2015, the first day of snow cover*, and loss of snow cover †

10.7717/peerj.3869/supp-4 Table S3 Mean size of home range overlap area of each individual among the intra- and inter-capture sites in each season and throughout the entire year

10.7717/peerj.3869/supp-5 Supplemental Information 1 Raw location data of the COAs shown in Figs. 2 and 3 COA location for each individual. COAs were calculated using seasonal home ranges. The X and Y coordinates (in UTM; m) of individual COAs are shown for each season. Seasons are labeled as winter 1 (first winter), summer, and winter 2 (second winter) with period defined via the NSD method (see the Methods section in the manuscript).

10.7717/peerj.3869/supp-6 Supplemental Information 2 Shapefile raw data of annual home range each individual for Fig. 4 Home range area of deer of Kushiro Wetland in a year each individual. The home range area was estimated by a 95% local convex hull using location data for a year.

10.7717/peerj.3869/supp-7 Supplemental Information 3 Shapefile containing the raw data of seasonal home ranges for each individual shown in Fig. S1 Seasonal home range area of Kushiro Wetland deer in (A) first winter, (B) summer, and (C) second winter for one year each individual. The home ranges were estimated by a 95% local convex hull.

10.7717/peerj.3869/supp-8 Supplemental Information 4 Raw data used to calculate the values in Table 3 Annual home range size (m^2^); home range in wetland (*m*^2^), amount of the annual home range comprised of wetland habitat (*m*^2^); home range outside wetland, amount of home range comprised of non-wetland habitat (*m*^2^), percent wetland (%), home range in wetland/annual home range size for each individual. These data are summarized in Table 3.

10.7717/peerj.3869/supp-9 Supplemental Information 5 Raw migration initiation data used to generate the values in Table S2 Migration initiation data for each individual exhibiting movement classified as migration.

10.7717/peerj.3869/supp-10 Supplemental Information 6 Raw seasonal migration distance data Spring and autumn migration distances for each individual exhibiting movement classified as seasonal migration. These data are summarized and shown in the manuscript. Two irregular migrants, which were estimated as they still have not reached the second winter habitat by the NSD method, are excluded.

## Additional Information and Declarations

### Field Study Permissions

The following information was supplied relating to field study approvals (i.e., approving body and any reference numbers): Permission for field study on government land was obtained from the Hokkaido Development Bureau. Approval Numbers: 68, 69 and 105.

### Data Availability

The following information was supplied regarding data availability: The raw data and code are included as Supplemental Files.
