# Supplementary figures and images for: Seasonal and year-round use of the Kushiro Wetland, Hokkaido, Japan by sika deer (Cervus nippon yesoensis)
Source: PeerJ. 2017 Oct 12;5:e3869. doi: 10.7717/peerj.3869 (PMC5641432; doi:10.7717/peerj.3869)

(A) First winter

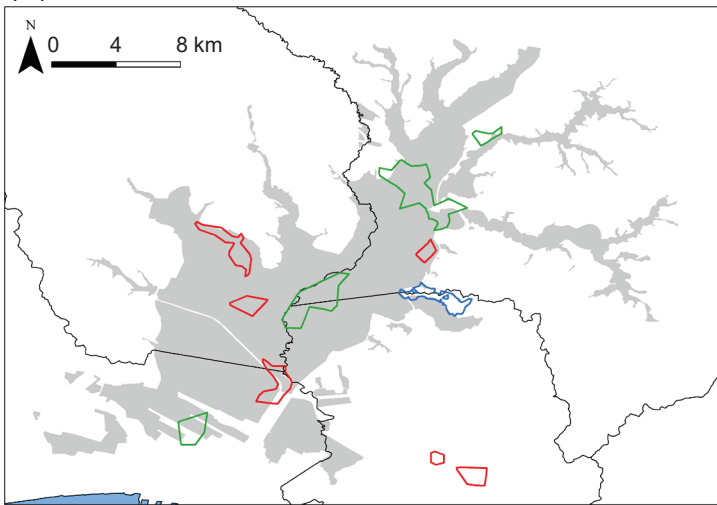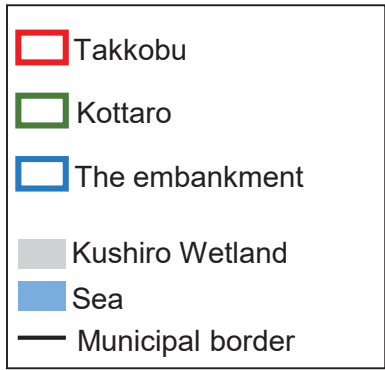

(B) Summer

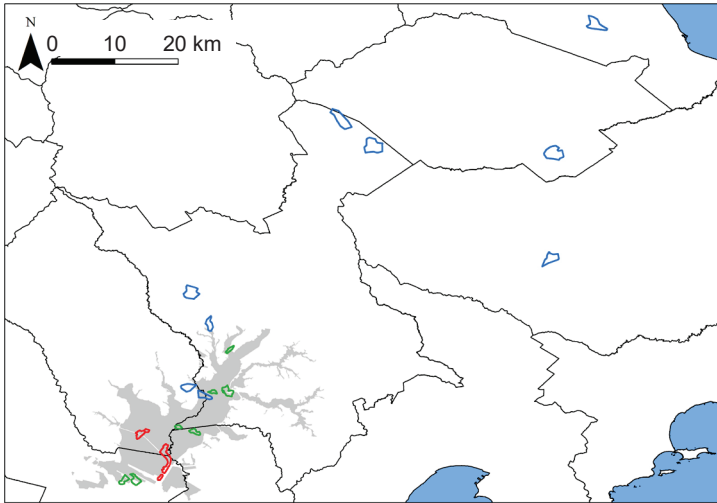

(C) Second winter

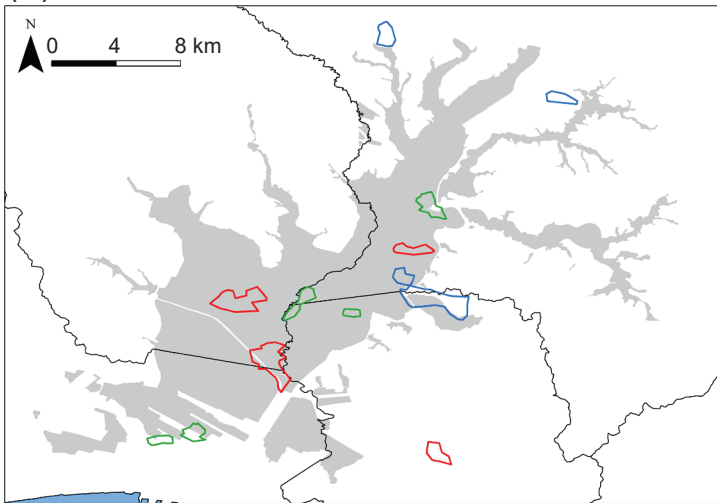

Supplement: Figure S1 — The home range area was estimated by all recorded locations of deer and determining the 95% local convex hull for them. The periods of the seasons were defined as well as the definition used in the COA calculation (see methods section) Red outline, home ranges of individuals captured at Takkobu; green outline, home ranges of individuals captured at Kottaro; blue outline, home ranges of individuals captured at embankment; shaded grey area, Kushiro Wetland; filled blue areas, sea; solid lines, municipality boundaries. [file peerj-05-3869-s001.pdf]
