# Supplementary material for: Seasonal and year-round use of the Kushiro Wetland, Hokkaido, Japan by sika deer (Cervus nippon yesoensis)
Source: PeerJ. 2017 Oct 12;5:e3869. doi: 10.7717/peerj.3869 (PMC5641432; doi:10.7717/peerj.3869)
Supplement: Table S1 [file peerj-05-3869-s002.docx]

| Capture site | Tag ID | Results from NSD | Results from Overlap | Final result |
| --- | --- | --- | --- | --- |
| Takkobu | 35285 | Migration | Migration | Migration |
| Takkobu | 36750 | Migration | No-return | Atypical |
| Takkobu | 36751 | Migration | No-return | Atypical |
| Takkobu | 36748 | Migration | Migration | Migration |
| Takkobu | 36755 | Migration | No-return | Atypical |
| Takkobu | 36754 | Migration | Migration | Migration |
| Takkobu | 36749 | Migration | No-return | Atypical |
| Takkobu | 36753 | Migration | Migration | Migration |
| The embankment | 36724 | Migration | Resident | Atypical |
| The embankment | 36725 | Migration | Migration | Migration |
| The embankment | 36726 | Migration | Resident | Atypical |
| The embankment | 36728 | Migration | Migration | Migration |
| The embankment | 36731 | Migration | Migration | Migration |
| The embankment | 36741 | Migration | No-return | Atypical |
| The embankment | 36727 | Resident | Resident | Resident |
| The embankment | 36732 | Nomadic | Resident | Atypical |
| Kottaro | 36736 | Migration | No-return | Atypical |
| Kottaro | 36740 | Dispersal | Resident | Atypical |
| Kottaro | 36733 | Migration | No-return | Atypical |
| Kottaro | 36738 | Resident | Migration | Atypical |
| Kottaro | 36735 | Migration | Resident | Atypical |
| Kottaro | 32532 | Dispersal | Migration | Atypical |
| Kottaro | 36737 | Dispersal | No-return | Dispersal |
| Kottaro | 36734 | Dispersal | No-return | Dispersal |
| Kottaro | 36739 | Resident | Resident | Resident |
|  |  |  |  |  |
