# Supplementary material for: Seasonal and year-round use of the Kushiro Wetland, Hokkaido, Japan by sika deer (Cervus nippon yesoensis)
Source: PeerJ. 2017 Oct 12;5:e3869. doi: 10.7717/peerj.3869 (PMC5641432; doi:10.7717/peerj.3869)
Supplement: Table S2 [file peerj-05-3869-s003.docx]

|  | | | | | | | |
| --- | --- | --- | --- | --- | --- | --- | --- |
| Year | Capture site | Spring migration  ± SD | Date of loss of snow cover | | Autumn migration  ± SD | Date of first snow cover | *n* |
| 2014 | Takkobu | May 5 | Apr 4 | Aug 26 | | Dec 11 | 1 |
|  |  |  |  |  | |  |  |
| 2015 | Takkobu | Apr. 14±4.0day | Apr 10 | Nov. 11±47.1day | | Nov 24 | 3 |
| 2015 | The embankment | March 27±3.6day | Apr 10 | Feb. 3±62.0day | | Nov 24 | 3 |
| *the first day that snow depth was less than 1 cm as measured at the Tsurui weather station; SD, standard deviation; †the first day that snow accumulation exceeded 1 cm as measured at the Tsurui weather station | | | | | | | |
