# Supplementary material for: Seasonal and year-round use of the Kushiro Wetland, Hokkaido, Japan by sika deer (Cervus nippon yesoensis)
Source: PeerJ. 2017 Oct 12;5:e3869. doi: 10.7717/peerj.3869 (PMC5641432; doi:10.7717/peerj.3869)
Supplement: Table S3 [file peerj-05-3869-s004.docx]

|  | Annual | |  | First winter | |  | Summer | |  | Second winter | |
| --- | --- | --- | --- | --- | --- | --- | --- | --- | --- | --- | --- |
|  | intra-capture sites | inter-capture sites |  | intra-capture sites | inter-capture sites |  | intra-capture sites | inter-capture sites |  | intra-capture sites | inter-capture sites |
| Takkobu | 48.0 | 0.3 |  | 54.9 | 0.0 |  | 20.0 | 1.8 |  | 47.7 | 1.1 |
| The embankment | 63.5 | 3.4 |  | 44.6 | 0.0 |  | 63.5 | 0.0 |  | 61.9 | 1.2 |
| Kottaro | 31.4 | 0.5 |  | 26.9 | 0.0 |  | 0.0 | 0.8 |  | 35.3 | 0.0 |
| Total | 47.7 | 1.3 |  | 65.0 | 0.0 |  | 34.2 | 0.8 |  | 48.3 | 0.6 |
